# Differentiation of Oligodendrocyte Precursor Cells from *Sox10*-Venus Mice to Oligodendrocytes and Astrocytes

**DOI:** 10.1038/s41598-017-14207-0

**Published:** 2017-10-26

**Authors:** Nobuharu Suzuki, Kaori Sekimoto, Chikako Hayashi, Yo Mabuchi, Tetsuya Nakamura, Chihiro Akazawa

**Affiliations:** 1 Department of Biochemistry and Biophysics, Graduate School of Health Care Sciences, Tokyo Medical and Dental University, Tokyo, Japan; 2 Department of Gastroenterology and Hepatology, Tokyo Medical and Dental University, Tokyo, Japan; 3 Department of Advanced Therapeutics for GI Diseases, Tokyo Medical and Dental University, Tokyo, Japan

## Abstract

Oligodendrocytes are well known as myelin-forming cells in the central nervous system (CNS). However, detailed mechanisms of oligodendrocyte differentiation and myelination are poorly understood, particularly due to the difficulty of the purification of murine oligodendrocyte precursor cells (OPCs). We have recently established a transgenic mouse line that expresses a fluorescent protein Venus under the promoter of Sox10, whose expression is restricted to OPCs and oligodendrocytes in the CNS. Here, we have characterized Venus-positive cells from the *Sox10*-Venus mouse brain for analyzing oligodendrocyte differentiation. We first purified Venus-positive cells from the postnatal day 0–2 brain by flow cytometry. Most of the Venus-positive cells expressed NG2, an OPC marker. After induction of differentiation, an increased population of galactocerebroside-positive oligodendrocytes and decrease of OPCs were observed in the Venus-positive culture. Furthermore, a time-lapse analysis showed that Venus-positive oligodendrocytes dynamically changed their morphology with highly branched cell processes during differentiation. In addition, we found that Venus-positive OPCs were able to differentiate to type II astrocytes. *In vivo*, OPCs and oligodendrocytes express Venus, and some of astrocytes were positive for Venus in the ventral cortex. Taken together, the *Sox10*-Venus mouse system is useful for analyzing differentiation and multipotency of murine OPCs.

## Introduction

Oligodendrocyte precursor cells (OPCs; also known as oligodendrocyte progenitor cells, polydendrocytes, or O-2A cells) are glial cells that possess the potential to give rise to oligodendrocytes in the central nervous system (CNS)^1,2^. OPCs generated from glial precursor cells differentiate into myelinating oligodendrocytes, which form consecutive layers of membrane surrounding the neuronal axons facilitating the propagation of the action potentials. During development, oligodendrocyte lineage cells undergo significant morphological changes by forming elaborated branching cell processes. OPCs differentiate into oligodendrocytes not only during the development of the CNS but also in cases of pathological demyelination in the mature CNS^1^. Since oligodendrocytes play a crucial role in dysmyelinating diseases, such as leukodystrophy and multiple sclerosis, the study of oligodendrocytes and their differentiation mechanisms is essential for the development of novel therapies.

Previous studies have shown that OPCs can differentiate into not only oligodendrocytes but also astrocytes and neurons, depending on the area of the brain and stages during CNS development. However, the majority of the OPC population differentiates to oligodendrocytes^3^. *In vitro*, OPCs differentiate to oligodendrocytes under serum-free culture conditions, while their differentiation can be directed towards astrocyte-lineage cells under high-serum culture conditions^4^. Classically, these astrocytic cells derived from OPCs are called type II astrocytes, which are distinguished from type I astrocytes derived from glial precursor cells^4^. However, the functional differences between type I and II astrocytes in physiological and/or pathological conditions have not yet been elucidated. Therefore, specific labeling/tracing of both OPCs and OPC-lineage cells, and purification of these cells for *in vitro* studies, are essential tools to unravel the disease and injury mechanisms in the CNS.

Although isolation of rat OPCs from the CNS has been previously established, it is still less efficient to obtain sufficient quantity and purity of mouse OPCs^5^. One of the reasons for the difference between these two species is the distinct expression pattern of cell surface markers. The monoclonal antibody A2B5, whose antigen is a ganglioside, is widely used for purification of rat OPCs. However, mouse OPCs can not be efficiently purified by this antibody, since the expression level of the ganglioside in mice is lower than that in rats^6,7^. Therefore, for mouse OPC purification, neural/glial antigen 2 (NG2), instead of A2B5, is useful as a cell surface marker^8^. However, NG2 is expressed in not only OPCs but also in pericytes adherent to capillaries^9^. Another marker PDGFRα is available for immunopanning of OPCs from mouse cortices^10^. This is a useful and established method, but the possibility exists that in general, antibodies used for sorting may affect the cells during culture or analysis^11^. This problem can be overcome by using a fluorescent protein expression system under an OPC/oligodendrocyte-specific promoter. Several transgenic mouse lines that express a fluorescence protein DsRed or GFP under the regulation of OPC genes *Cspg4* and *Pdgfra*, respectively, have been developed to analyze the fate of oligodendrocyte lineage cells^12–14^. In this study, we introduce a purification and culture method of OPCs from the transgenic mouse line *Sox10*-Venus, in which the intense fluorescence protein Venus is expressed under the promoter of the *Sox10* gene^15^. Sox10, a high-mobility-group transcriptional regulator, is required for myelin gene expression^16^. In the CNS, Sox10 expression is elevated during development of glial precursor cells into OPCs, and its expression is persistent throughout oligodendrocyte differentiation and maturation^16^. Also, the fluorescence of Venus is more intense than that of DsRed and GFP^17^, and may be useful for the OPC differentiation analysis, particularly for the analysis of process formation during the differentiation. We have investigated the oligodendrocyte differentiation by following the cell fate of *Sox10*-Venus positive cells. We here propose that *Sox10*-Venus mice could be a useful tool for purifying and analyzing oligodendrocyte lineage cells.

## Results

### Antigenic phenotype of *Sox10*-Venus positive cells after purification using flow cytometer

**Figure 1:**
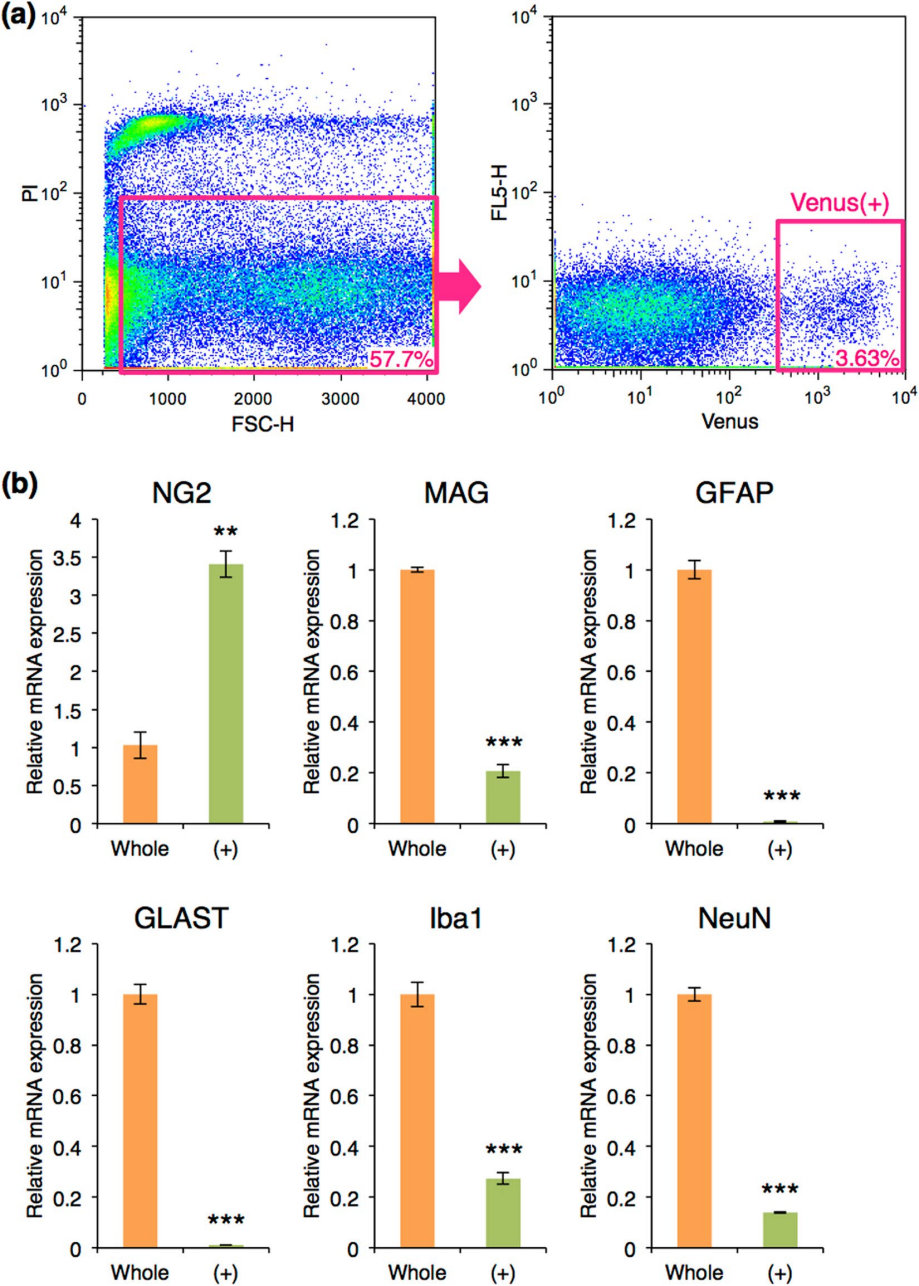
Cell sorting of Venus (+) cells using flow cytometry. (**a**) Flow cytometry analysis of Venus (+) cells. Cells positive for propidium iodide were removed as dead cells (pink box in the left panel), and cells with the strong intensity of Venus were collected as Venus (+) cells (pink box in the right panel). The numbers in pink indicates the fraction of each cell population in the flow cytometry. (**b**) Quantitative RT-PCR of glial and neural markers in whole brain cells before cell sorting and Venus (+) cells. The mRNA expression of each gene was normalized using β-actin mRNA expression. The normalized expression of each gene in whole brain cells was set as 1.0. Whole: whole brain cells before cell sorting, orange bar; (+): Venus (+) cells, green bar; Error bars, s.e.m. (**p < 0.01, ***p < 0.001, *t* test).

In order to analyze whether Venus-positive cells are OPCs and their differentiation, we used *Sox10*-Venus mouse brains at postnatal day (P) 0–2, when OPCs are enriched but not yet differentiated to oligodendrocytes, for isolation of Venus-positive cells by flow cytometer. Propidium iodide-positive cells and cell debris were removed (Fig. 1a: left panel). Cell populations showing high Venus intensity [Venus (+)] were collected (Fig. 1a: right panel). Quantitative RT-PCR using cells after the cell sorting revealed that Venus (+) cells highly expressed an OPC marker NG2, but not markers for oligodendrocytes: myelin associated glycoprotein (MAG), astrocytes: glial fibrillary acidic protein (GFAP) and glutamate/aspartate transporter (GLAST), microglia: ionized calcium-binding adapter molecule 1 (Iba1), or neurons: neuronal nuclei (NeuN), compared with control whole cells without cell sorting (Fig. 1b).

**Figure 2:**
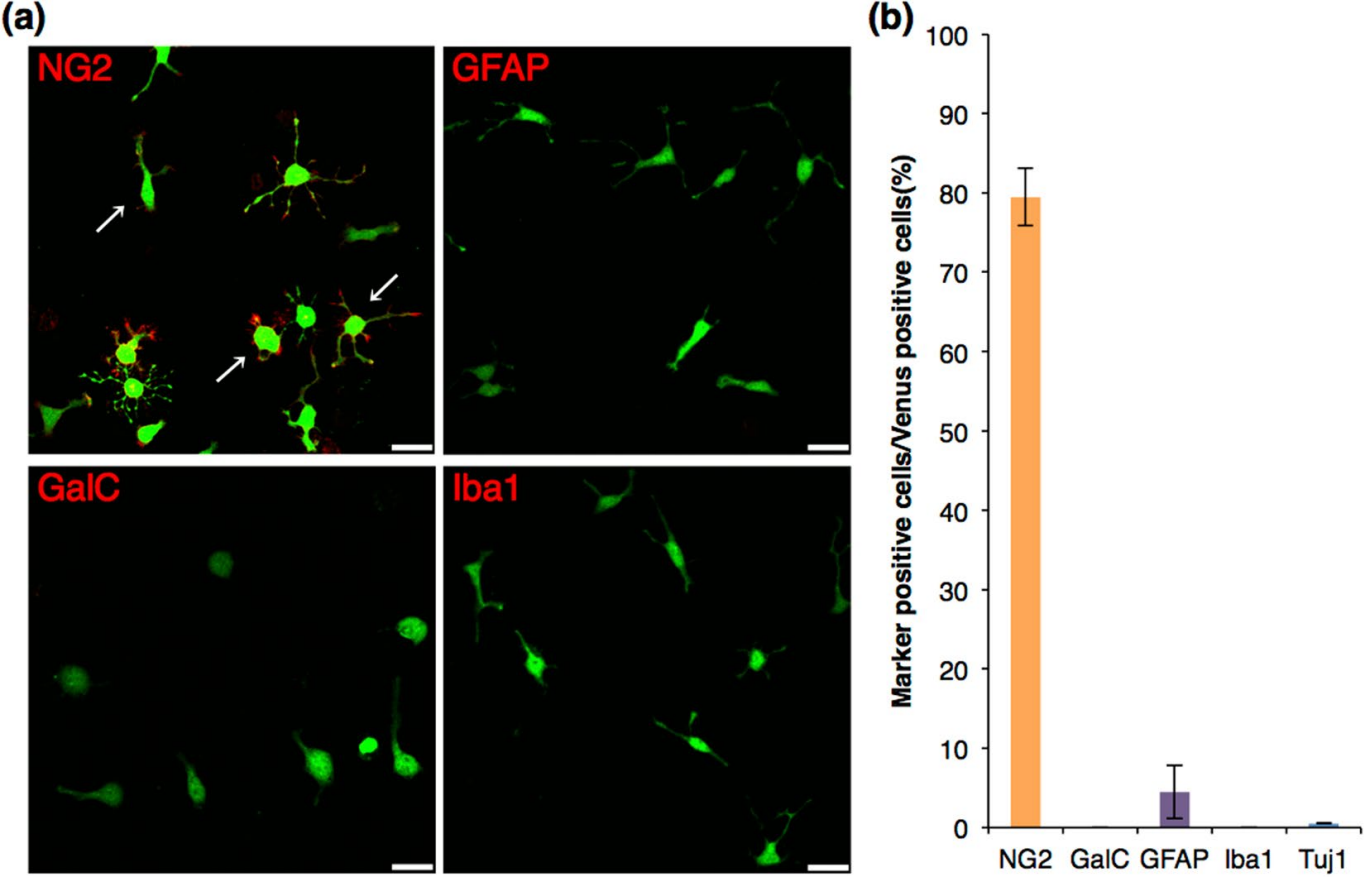
Immunocytochemistry of glial and neuronal markers in Venus (+) cells before induction of differentiation. (**a**) Confocal images of the immunocytochemistry before induction of differentiation. Venus fluorescence is shown in green, and each glial marker is shown in red. Scale bar, 25 µm. Venus (+) cells expressed NG2, showing typical cell morphology of OPCs in culture (arrows). (**b**) Cell count analysis showing cell population of Venus (+) cells expressing each marker. Error bars, s.e.m.

To determine the morphology and characteristics of Venus (+) cells, cells were cultured for 1 day in Proliferation medium. Most of the Venus (+) cells had round cell body with several primary processes, which resemble the typical morphology of OPCs in culture (Fig. 2a: arrows), showing immunoreactivity for NG2 on cytomembrane (Fig. 2a). Most of the Venus (+) cells were positive for NG2 (79 ± 3.6%), and a small population of GFAP-positive cells was observed (4.5 ± 3.4%) (Fig. 2b). Other cell-types, such as galactoceramide (GalC)-positive oligodendrocytes, Iba1-positive microglia, and Tuj1-positive neurons, were not present (Fig. 2b). In addition, Venus (+) cells were detectable by either anti-PDGFRα antibody or A2B5 antibody (Supplementary Figure S1a). Furthermore, most of the Venus (+) cells were positive for Ki67 and/or BrdU (Supplementary Figure S2), suggesting that Venus (+) cells under this condition are proliferative, which is one of the characteristics of OPCs. These results indicated that OPCs were enriched in the Venus (+) population. These observations showed that OPCs can be sorted by the intensity of the Venus fluorescence from the *Sox10*-Venus mouse brain at the neonatal stage.

### Differentiation of *Sox10*-Venus positive cells into oligodendrocytes

**Figure 3:**
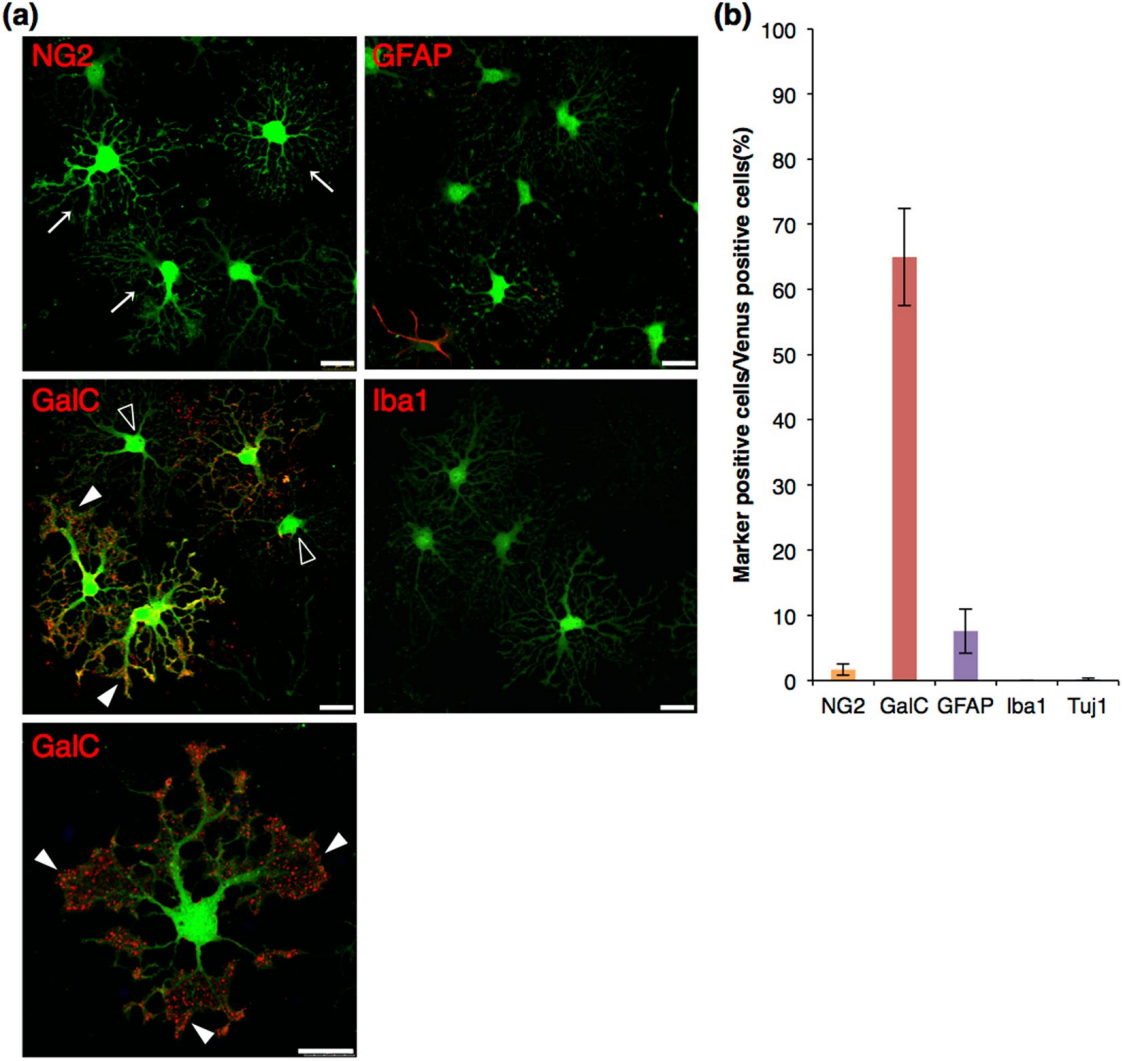
Immunocytochemistry of glial and neuronal markers in Venus (+) cells after oligodendrocyte differentiation. (**a**) Confocal images of the immunocytochemistry after oligodendrocyte differentiation. Venus fluorescence is shown in green, and each glial marker is shown in red. Venus (+) OPCs formed highly branched cell processes after 3 days of culture in Serum-free differentiation medium (arrows). These cells expressed GalC, showing sheet-like structures in their cellular processes (closed arrowheads). GalC-negative cells, whose morphology was likely differentiating OPCs to oligodendrocytes, were observed (opened arrowheads). Scale bar, 25 µm. (**b**) Cell count analysis showing cell population of Venus (+) cells expressing each marker. Error bars, s.e.m.

To assess the functional properties of Venus (+) OPCs, we evaluated the differentiation potential of Venus (+) cells *in vitro*. For oligodendrocyte differentiation assay, Venus (+) cells were cultured in Serum-free differentiation medium for 3 days. After induction of differentiation, OPCs extended their cell processes and formed numerous branches in the processes (Fig. 3a: arrows). OPCs underwent terminal differentiation into mature oligodendrocytes by losing the expression of NG2, and began to express myelin markers, such as GalC. As shown by immunocytochemistry, NG2-positive cell number dropped drastically to 1.7 ± 0.9% (Fig. 3b), compared with the percentage before the induction of differentiation (79 ± 3.6%) (Fig. 2b), while GalC-positive cell number increased up to 65 ± 7.5% (Fig. 3b). Some of GalC-positive cells possessed sheet-like structures in their cell processes, which are required for myelin formation (Fig. 3a: closed arrowheads). There were also GalC-negative cells, whose morphology looked like differentiating OPCs into oligodendrocytes (Fig. 3a: opened arrowheads). These GalC-negative cells were probably in between NG2-positive OPCs and GalC-positive oligodendrocytes. Further, a slight increase of GFAP-positive cells was observed (7.6 ± 3.4%) (Fig. 3b), relative to the population before differentiation (4.5 ± 3.4%) (Fig. 2b). No Iba1- or Tuj1-positive cells were present (Fig. 3b). These results suggested that Venus (+) OPCs were differentiated into oligodendrocytes under the serum-free condition.

### Differentiation of *Sox10*-Venus positive OPCs into astrocytes in the presence of high concentration of serum

**Figure 4:**
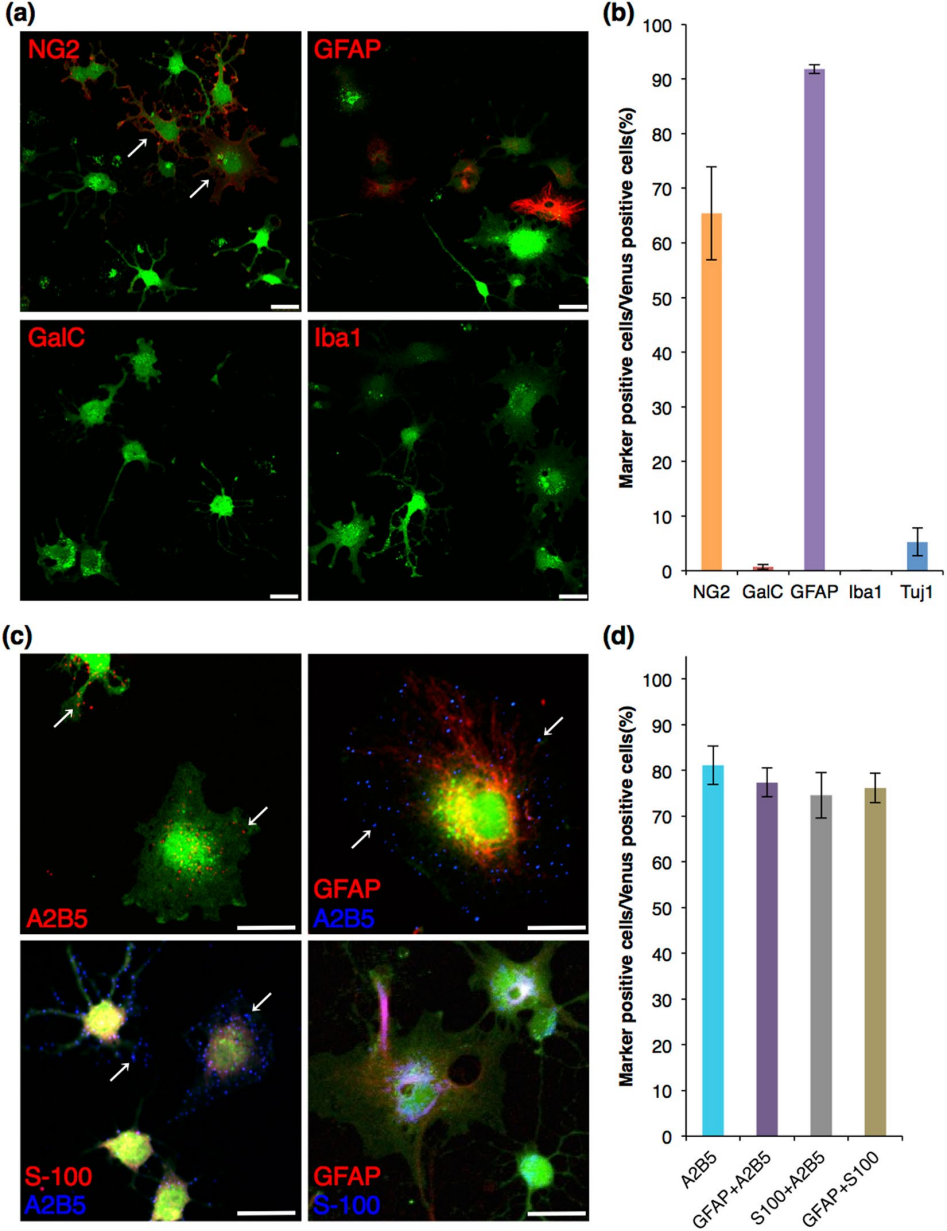
Immunocytochemistry of glial and neuronal markers in Venus (+) cells after astrocytic differentiation. (**a**,**c**) Confocal images of the immunocytochemistry after astrocytic differentiation. Venus fluorescence is shown in green, and each glial marker is shown in red or blue. Venus (+) OPCs developed into flat shaped cells with short primary cell processes (A: arrows). Most of Venus (+) cells expressed A2B5 with GFAP or S-100, or GFAP with S-100. Arrow: punctated staining of A2B5 on Venus (+) cell; Scale bar, 25 µm. (**b**,**d**) Cell count analysis showing cell population of Venus (+) cells expressing each marker. Error bars, s.e.m.

OPCs possess the ability to generate type II astrocytes as well as oligodendrocytes *in vitro* ^4^. It was reported that serum-containing differentiation medium, containing 20% fetal bovine serum (FBS) is suitable for induction of astrocytic differentiation^18^. Thus, we examined the characteristics of purified Venus (+) OPCs cultured in Proliferation medium for 1 day, and then cultured in Serum-containing differentiation medium thereafter. After 3 days culture in Serum-containing differentiation medium, 92 ± 0.8% of the cells expressed GFAP, and many of these cells were NG2-positive and changes in cell morphology were observed (Fig. 4a,b). Less than 1% of the cells expressed GalC (0.7 ± 0.5%) (Fig. 4a,b). None of the cells were positive for Iba1, whereas a small population was positive for Tuj1 (6.3 ± 2.0%) (Fig. 4a,b). After the astrocytic differentiation, Venus (+) OPCs formed lots of primary cell processes and developed into flat and stellate-shaped cells with large nuclei (Fig. 4a: arrows). The Venus intensity slightly weakened, although NG2 staining on protrusions radiating out from the large and flat cell body was observed (Fig. 4a: arrows). Some cells showed OPC-like cell morphology, although these cells did not differentiate into mature oligodendrocytes under this culture condition (Fig. 4a,b). These observations indicated that Venus (+) OPCs differentiated into astrocytic cells. To determine whether these GFAP-positive Venus (+) cells were type II astrocytes, A2B5 antibody, a marker for type II astrocytes, was used for the immunocytochemistry analysis. Punctuated staining of ganglioside, an antigen for A2B5, was observed in 81 ± 4.2% out of the total cells (Fig. 4c: arrows, 4d). Further, most of A2B5-positive cells immunoreacted with GFAP and S-100, another marker for astrocytes [Fig. 4c,d: GFAP/A2B5 (77 ± 3.2%), S-100/A2B5 (75 ± 5.0%)]. Also, most of GFAP-positive cells expressed S-100 (76 ± 3.2%) (Fig. 4c,d). These immunocytochemistry results showed that Venus (+) OPCs were differentiated into type II astrocytes.

### Time-lapse analysis of *Sox10*-Venus positive cells

**Figure 5:**
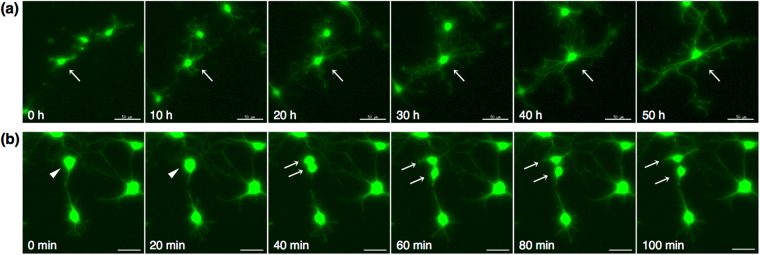
Time-lapse analysis of Venus (+) cells. (**a**) Differentiation of Venus (+) OPCs. *In vitro* time-lapse images were captured to follow the process formation of Venus (+) oligodendrocytes after induction of differentiation. Images every 10 hours are representatively indicated. Arrow: differentiating OPC with branched process formation; Scale bar, 50 µm. (**b**) Cell division of Venus (+) OPCs. Representative cell division images are shown every 20 minutes. Arrowhead: OPC before cell division; Arrows: OPCs after cell division; Scale bar, 30 µm.

Time-lapse analysis was performed to observe the morphological change of purified Venus (+) OPCs. After 1 day-culture in Proliferation medium, the medium was changed to Serum-free differentiation medium, and time-lapse images were captured every 20 minutes. During the observation period, OPCs underwent extensive morphological remodeling when they differentiated from bipolar OPCs into oligodendrocytes. Mature oligodendrocytes extended complex and highly branched processes (Fig. 5a Supplementary Video 1). Cell division was also observed in some cells (Fig. 5b, Supplementary Video 2).

### *Sox10*-Venus positive cells during postnatal development in the brain tissues

**Figure 6:**
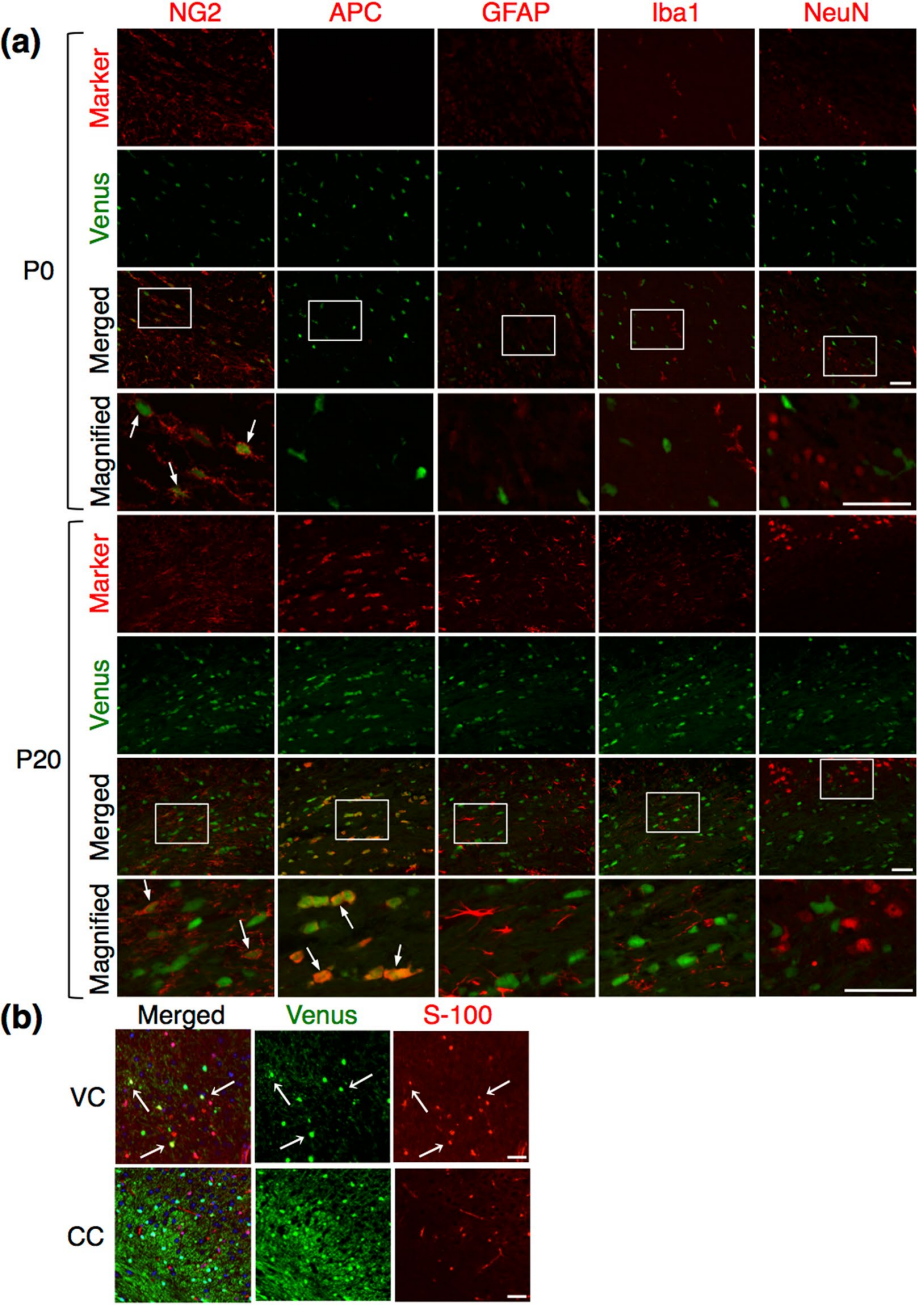
Immunohistochemistry of glial and neuronal markers in Venus (+) cells in the brain tissues. (**a**) Immunohistochemistry in the corpus callosum of the P0 and P20 posterior forebrain, showing the distribution of Venus positive cells (green) with each marker (red). Arrows: dual-positive cells of Venus with a marker; Scale bar: 50 µm. (**b**) Immunohistochemistry of S-100 in the ventral cortex (VC) and the corpus callosum (CC) at 12 months. Dual-positive cells of Venus with S-100 were observed in the ventral cortex (arrows); Scale bar: 50 µm.

We further analyzed Venus (+) cells in the brain tissues by immunohistochemistry. At P0, Venus (+) cells were distributed broadly in the brain tissues, and a slightly higher signal of Venus was observed in the corpus callosum tissue, compared to other regions (Supplementary Figure S3). Immunostaining analysis in the corpus callosum revealed that Venus (+) cells expressed NG2 and PDGFRα, but not Adenomatous Polyposis Coli (APC), a marker for mature oligodendrocytes, GFAP, Iba1, or NeuN, at this stage (Fig. 6a and Supplementary Figure S1b). APC was rarely detectable in this tissue, since OPCs are not yet differentiated to mature oligodendrocytes at P0. This result suggested that Venus (+) cells at P0 were OPCs. We performed the same analysis using the tissue sections from P20, when myelination intensively occurs. Strong Venus fluorescence was observed in the corpus callosum and the striatum tissues (Supplementary Figure S3). We found that Venus (+) cells were positive for APC, in addition to NG2, but negative for GFAP, Iba1, or NeuN (Fig. 6a). These data indicated that Venus (+) cells corresponded to oligodendrocyte-lineage cells, including OPCs and oligodendrocytes, as expected due to the endogenous Sox10 expression pattern^16^.

We also investigated whether Venus was expressed in astrocytes in the ventral cortex as reported previously^13^. Venus (+) cells positive for GFAP were hardly detected not only in the corpus callosum (Fig. 6a) but also in the ventral cortex, the dorsal cortex, and the striatum (data not shown). However, a broader marker for astrocytes, S-100, was positive in some of Venus (+) cells in the ventral cortex (Fig. 6b), as well as in the striatum (data not shown), but not in the corpus callosum (Fig. 6b). These evidences showed that Venus (+) cells were OPCs and were able to differentiate into oligodendrocytes and astrocytes, in both the brain tissues and the *in vitro* culture. All together, the results presented in this study showed that *Sox10*-Venus mice are useful for both *in vitro* and *in vivo* studies of OPCs, such as differentiation and morphological analyses.

## Discussion

In this study, we report a mouse OPC purification and culture method using *Sox10*-Venus mice. We showed that Venus (+) cells from P0-2 brains were OPCs and differentiated to either oligodendrocytes or astrocytes, depending on the culture conditions. Further, we were able to observe the formation of cell processes during the OPC differentiation to oligodendrocyte by time-lapse analysis.

Several transgenic mouse lines with a reporter system of a fluorescent protein under an oligodendrocyte lineage cell-specific promoter have been recently reported for OPC purification and culture experiments. Zhu *et al*. identified the characteristics of OPCs purified from NG2DsRedBAC transgenic mice that express DsRed in NG2-expressing cells. After cell sorting with a fluorescence of DsRed at P3, almost 100% of purified DsRed (+) cells were positive for NG2, and about 20% of the total cells differentiated to oligodendrocytes positive for O1 after induction of differentiation^13^. In addition, more than 85% of total cells were still positive for NG2 cells^13^. In the present study, we succeeded in obtaining differentiated GalC-positive oligodendrocytes more efficiently (>64%) from *Sox10*-Venus OPCs (Fig. 3). Moreover, the percentage of NG2 positive cells after differentiation culture was less than 2% out of total cells (Fig. 3). In addition to the NG2DsRedBAC line, the GFP reporter system of PDGFRα, another OPC marker, was utilized for purification and characterization of OPCs in culture^12^. However, using these systems, it is unable to perform a time-lapse experiment with these fluorescent proteins, since activity of either NG2 or PDGFRα promoter is dispersed during the differentiation to oligodendrocytes. Whereas the *CNP*-EGFP mouse line is also useful for studies of oligodendrocyte lineage cells, combination with cell sorting using anti-NG2 antibody is needed for purification of OPCs from this mouse line, as the CNP promoter activity begins just after the onset of the differentiation^19^. In contrast, Sox10 is continuously expressed during the stages from OPCs to oligodendrocytes^16^. In the present study, we were able to evaluate the OPC differentiation and to observe cell process formation and cell division in the culture by time-lapse analysis (Figs 2–5, Supplementary Video 1, 2). Recently, Tripathi *et al*. created *Sox10*-GFP/tdTom dual reporter mice and their group purified either GFP- and tdTomato-positive OPCs from the ventral and dorsal regions in the brain, respectively, by flow cytometer^20,21^. They found that there were differences in OPC migration and differentiation capacities between the ventral and dorsal area-derived OPCs from the adult brain. However, they did not stain the cells with other glial or neuronal markers, neither they induced their differentiation to astrocytes nor carried out time-lapse analysis, which were analyzed in this study (Figs 2–5, Supplementary Video 1, 2). Given these observations and evidences, we provide here a useful method for analyzing OPC differentiation to oligodendrocytes *in vitro* using *Sox10*-Venus mice. Further, the Venus fluorescence is more intense than that of other fluorescent proteins, including DsRed, GFP, and tdTomato, which were used in the previous studies^12–14,17,20,21^. Particularly, the intense fluorescence of *Sox10-*Venus was very useful and reliable to observe the fine structures of cell protrusions during the processes formation (Fig. 5, Supplementary Video 1, 2).

OPCs have the potential to differentiate not only to oligodendrocytes but also to type II astrocytes *in vitro*. Culturing under high concentration of FBS, fetal calf serum, or the addition of bone morphogenetic proteins, induces OPCs differentiation into type II astrocytes^19,22^. Type II astrocytes are differentiated from OPCs and express specific antigens, such as GFAP, S-100, and the ganglioside for A2B5. In this study, we demonstrated an efficient differentiation of type II astrocytes from *Sox10*-Venus OPCs in the presence of 20% FBS, and found that most of the astrocytic cells from Venus (+) OPCs express NG2 (Fig. 4), as previously reported^13^, although NG2 is a marker for OPCs. We can postulate that type II astrocytes maintain NG2 expression, in addition to a significant increase of astrocytic marker expression. The differences in protein expression between type I and II astrocytes may reflect their distinct functional properties.

In our previous work, we demonstrated that Venus is specifically expressed in oligodendrocyte-lineage cells in the spinal cord of *Sox10*-Venus mice at embryonic day 15.5 and 8 weeks after birth^15^. In the present study, Venus (+) cells expressed NG2 at P0, and NG2 or MBP at P20 in the corpus callosum of the *Sox10*-Venus mouse brain (Fig. 6). This expression pattern of Venus is consistent with that of endogenous Sox10 during OPC differentiation to oligodendrocytes^16^. Further, S-100- and Venus-double positive cells were observed in the ventral cortex, but not in the corpus callosum, of the *Sox10*-Venus brain, while double positive cells for GFAP and Venus were rarely detected (Fig. 6). Previously, *in vivo* cell fate mapping of OPCs has been carried out. Zhu *et al*. reported that S-100-positive astrocytes derived from OPCs reside in the ventral cortex more than in the corpus callosum of the NG2CreBAC:Z/EG transgenic mice, and even smaller number of GFAP-positive astrocytes derived from OPCs was found, compared with OPC-derived S-100-positive astrocytes^13^. This agrees with our present data. Also, some studies demonstrate that OPCs give rise to neurons as well as oligodendrocytes and astrocytes, but others denied that^2,23^. In our system, none of the neurons expressed Venus in the *Sox10*-Venus brain (Fig. 6). These controversial evidences among the reported data, regarding neurogenesis from OPCs, is probably due to differences of the reporter gene regulation systems and/or of timing or area for the analyses. More detailed studies are needed to elucidate the mechanism.

In summary, we established an efficient purification method of OPCs, which were differentiated into oligodendrocytes and type II astrocytes, using *Sox10*-Venus mice. In the *Sox10*-Venus brain, Venus (+) OPCs, oligodendrocytes, and astrocytes were found. Analyses using the *Sox10*-Venus mouse system will facilitate a better understanding of developmental and pathological function of OPCs *in vivo* and of cellular and molecular OPC function *in vitro*.

## Methods

### Animals

P0-2 pups from the previously reported *Sox10*-Venus mice^15^ were euthanized by decapitation as described in the International Animal Care and Use Committee and the previous report^5^, and then, they were used for the experiments. All procedures for animal experiments were approved by the Tokyo Medical and Dental University Animal Care and Use Committee (Protocol number: 0170282 C) and the Juntendo University School of Medicine Animal Care and Use Committee (Institutional review board No. 270050). All methods were conducted in strict accordance with the approved guidelines of the institutional animal care committees.

### Primary cell preparation

Primary mixed cell cultures were prepared from the brain of P0-2 mouse pups. The whole brain was dissected out and the meninges were removed. Then, the brains were diced into 1 mm^3^ chunks in a dish with ice-cold Leibovitz’s L-15 medium (Life Technologies) and enzymatically dissociated with 10 ml of Papain/DNase I solution containing: 40 mg of α-d-Glucose (SIGMA-ALDRICH), 4 mg of bovine serum albumin (SIGMA-ALDRICH), 4 mg of l-cysteine (SIGMA-ALDRICH), 2.5 µl/ml Papain (Worthington), 5 µl/ml DNase I (SIGMA-ALDRICH) in phosphate buffered saline (PBS). After an incubation for 20 minutes at 37 °C, the cells were collected by centrifugation in a swinging bucket at 100 *g* for 5 minutes. The supernatant was removed and Dulbecco’s modified Eagle’s medium (DMEM; Life Technologies), supplemented with 10% FBS (Thermo Fisher Scientific), as well as sodium pyruvate (SIGMA-ALDRICH), l-glutamine (Life Technologies), and 100 units/ml penicillin and 100 µg/ml streptomycin (Life Technologies) were added. The pellet was dissociated, and the tissue suspension was filtered through a 70 µm nylon cell strainer (Falcon).

### Isolation of *Sox10*-Venus positive cells using flow cytometry

The cell suspension was collected in a 5 ml tube with cell-strainer cap (Falcon) and was labeled with propidium iodide (SIGMA-ALDRICH) for 10 minutes to remove cell debris and dead cells. Cell sorting was performed using JSAN flow cytometer (Bay bioscience) or MoFlo flow cytometer (Beckman). Cells were collected in a tube containing DMEM supplemented with 10% FBS, sodium pyruvate, l-glutamine, and penicillin/streptomycin.

### Cell culture and differentiation of *Sox10*-Venus positive cells

Sorted cells were plated in Poly-d-Lysine 8-Well Culture Slides (BD Biosciences) at 3.0 × 10^4^ cells per well and incubated under 5% CO_2_ at 37 °C. Cells were cultured in Proliferation medium [Basal chemically defined medium (BDM) supplemented with 0.5% FBS, and penicillin/streptomycin]. BDM was prepared by adding 0.1% BSA, 4 mM l-glutamine, 1 mM sodium pyruvate, 30 nM sodium selenite (SIGMA-ALDRICH), 10 nM hydrocortisone (SIGMA-ALDRICH), 10 nM d-biotin (SIGMA-ALDRICH), 50 µg/ml Apo-transferrin (SIGMA-ALDRICH), 5 µg/ml insulin (SIGMA-ALDRICH), and penicillin/streptomycin in DMEM. There was no difference in proliferation and differentiation of Venus (+) OPCs between 0.5% FBS- and 10 ng/ml of PDGF-AA/basic-FGF-contained media during the 1-day culture. One day after plating, the medium was changed to serum-free differentiation medium [BDM supplemented with 40 ng/ml triiodo-thyronine (SIGMA-ALDRICH), 1 ng/ml ciliary neurotrophic factor (Peprotech), 50 ng/ml N-acetyl-l-cysteine (SIGMA-ALDRICH)] and cultured for 3 days. For astrocytic differentiation, cell medium was changed to Serum-containing differentiation medium [BDM containing 20% FBS and penicillin/streptomycin] for 3 days.

### Immunostaining

The cells or frozen tissue sections were fixed with 4% paraformaldehyde (PFA) in PBS (Wako) for 15 minutes at room temperature, and permeabilized with 0.1% Triton X-100 (SIGMA-ALDRICH) for detection of intracellular proteins and a membrane marker NG2. To detect another membrane marker GalC, the permeabilization step was omitted. After blocking with Power Block Universal Blocking Reagent (BioGenex Laboratories) for 1 hour at room temperature, cells were incubated with primary antibodies overnight at 4 °C. The primary antibodies were as follows: rabbit anti-NG2 (1:250; Millipore), mouse anti-GalC (1:250; Millipore), mouse anti-A2B5 (1:300; abcam), rabbit anti-GFAP (1:1000; Thermo Scientific), mouse anti-S100 (1:1000; SIGMA-ALDRICH), mouse anti-APC (1:20; Millipore), mouse anti-NeuN (1:100; Millipore), and rabbit anti-Iba1 (1:250; Wako). After washing, the cells were labeled with secondary antibodies for 45 minutes at room temperature. The secondary antibodies were as follows: rabbit/mouse IgG-Alexa 594 (Life Technologies), mouse IgG- and mouse IgM-Alexa 647 (Life Technologies). The samples were mounted with Vectashield containing DAPI (Vector Laboratories).

### Analysis of cell morphology and cell count

For antigenic phenotyping, positive cells for each antigen were counted and expressed as a percentage out of all Venus positive cells. Confocal microscopy images were obtained using a Leica TCS-SP5 confocal laser scanning microscope (Leica Microsystems), and all confocal settings were set to the same parameters for each experiment. Metamorph software was used for analysis, and at least three independent sorting experiments were analyzed. The numbers were expressed as means ± standard error.

### RT-PCR

Total RNA was isolated from 6 wells of the primary *Sox10*-Venus cell culture in the poly-_D_-Lysine 8-Well Culture Slide using TRI-Reagent (SIGMA-ALDRICH) as described previously^24^. Seventy-five μg of total RNA was reverse-transcribed into cDNA using SuperScript III Reverse Transcriptase (Life Technologies) and Oligo dT Primer (Life Technologies). For quantitative RT-PCR, cDNA was amplified for initial denaturation at 95 °C for 20 seconds, and then 40 PCR cycles of 95 °C for 3 seconds, 60 °C for 30 seconds, using Fast SYBR Green Master Mix (Applied Biosystems), with the following gene-specific primers; NG2: 5′-TCAAAACTTCAGCGTTCCCG-3′ (forward), 5′-CAAAGGCGTCTGTCTGTGTCTCAC-3′ (reverse); MAG: 5′-GCCTTTGCCATCCTGATTGC-3′ (forward), 5′-TCTGAGTGGGAATAACTGAGGTCC-3′ (reverse); GFAP: 5′-CCACCAAACTGGCTGATGTCTAC-3′ (forward), 5′-CCTTTCTCTCCAAATCCACACG-3′ (reverse); GLAST: 5′-CGCAGGGAAGATTGTTGAGATG-3′ (forward), 5′-TGAAAGTGATGGGTAGGGTGGC-3′ (reverse); NeuN: 5′-TCTCTTGTCCGTTTGCTTCCAG-3′ (forward), 5′-TCCGATGCTGTAGGTTGCTGTG-3′ (reverse); Iba1: 5′-GTCCTTGAAGCGAATGCTGG-3′ (forward), 5′-CATTCTCAAGATGGCAGATC-3′ (reverse); β-actin: 5′-ATTGCTGACAGGATGCAGAA-3′ (forward), 5′-TAGAGCCACCAATCCACACAG-3′ (reverse).

### Time-lapse imaging

*In vitro* time-lapse images were captured to follow the cell processes formation after induction of differentiation. Time-lapse images were captured using a Delta Vision system (Applied Precision) equipped with an inverted microscope IX-70 (Olympus) with a 10× objective in temperature-controlled chamber (37 °C) in which a humidified premixed gas (5% CO2 and 95% air) was infused. Z-stack images were collected every 20 minutes for 50 hours.

### Frozen tissue sections

Mice were deeply anesthetized with sodium pentobarbital (30 mg/kg) and perfused transcardially with PBS and then with 4% PFA in PBS (Wako). Brain tissues were post-fixed with 4% PFA in PBS overnight at 4 °C. After fixation, tissues were stored in 15% sucrose solution in PBS at 4 °C overnight, and then in 30% sucrose solution in PBS overnight at 4 °C. Tissues were embedded with OCT compound (Sakura Finetek). The embedded tissues were sectioned using a cryostat (Leica) and immunohistochemistry was performed as described above.

### Data availability

All the data are presented in the manuscript as figures or additional files.

## Electronic supplementary material

Supplementary Video 1

Supplementary Video 2

Supplementary Figures
