# Supplementary material for: Differentiation of Oligodendrocyte Precursor Cells from Sox10-Venus Mice to Oligodendrocytes and Astrocytes
Source: Sci Rep. 2017 Oct 26;7:14133. doi: 10.1038/s41598-017-14207-0 (PMC5658394; doi:10.1038/s41598-017-14207-0)

SUPPLEMENTAL INFORMATION

Differentiation of Oligodendrocyte Precursor Cells from *Sox10*-Venus Mice to Oligodendrocytes and Astrocytes

Nobuharu Suzuki[1¶], Kaori Sekimoto[1¶], Chikako Hayashi[1], Yo Mabuchi[1], Tetsuya Nakamura[2], and Chihiro Akazawa[1*]

[1]Department of Biochemistry and Biophysics, Graduate School of Health Care Sciences, Tokyo Medical and Dental University, Tokyo, Japan, [2]Department of Gastroenterology and Hepatology, Tokyo Medical and Dental University, Tokyo, Japan/Department of Advanced Therapeutics for GI Diseases, Tokyo Medical and Dental University, Tokyo, Japan

* Corresponding author

E-mail: c.akazawa.bb@tmd.ac.jp (CA)

¶ These authors equally contributed to this work.

Supplementary Video 1: Time-lapse analysis of cell process formation of differentiating Venus (+) cells

Supplementary Video 2: Time-lapse analysis of cell division of Venus (+) cells

Supplementary Figure S1: PDGFRα and A2B5 staining in Venus (+) cells

Supplementary Figure S2: Staining for proliferation markers in Venus (+) cells before induction of differentiation

Supplementary Figure S3: Distribution of Venus (+) cells in the brain

Supplementary Video 1. Time-lapse analysis of cell process formation of differentiating Venus (+) cells. The 30 seconds-movie was created using Z-stack images collected every 20 minutes for 50 hours, as described in Methods.

Supplementary Video 2. Time-lapse analysis of cell division of Venus (+) cells. The 30 seconds-movie was created using Z-stack images collected every 20 minutes for 100 minutes.

Supplementary Figure S1. PDGFRα and A2B5 staining in Venus (+) cells. (**a**) Immunocytochemical images of purified Venus (+) cells before induction of differentiation. Venus fluorescence is shown in green, and each glial marker is shown in red. Scale bar, 50 μm. (**b**) Immunohistochemistry in the corpus callosum of the P0 posterior forebrain, showing the distribution of Venus positive cells (green) with PDGFRα (red). Scale bar: 50 μm.

Supplementary Figure S2. Staining for proliferation markers in Venus (+) cells before induction of differentiation. (**a**) Immunocytochemistry of Ki67 and BrdU before induction of differentiation. Venus fluorescence is shown in green, and each marker is shown in red. Scale bar, 25 μm. (**b**) Cell count analysis showing percentage of proliferative Venus (+) cells. Error bars, s.e.m.

Supplementary Figure S3. Distribution of Venus (+) cells in the brain. Fluorescence images of Venus (green) and DAPI (blue) on coronal sections of posterior brains at P0 and P20. CC: corpus callosum; ST: striatum; Scale bars: 1 mm.

Supplementary Figure S1

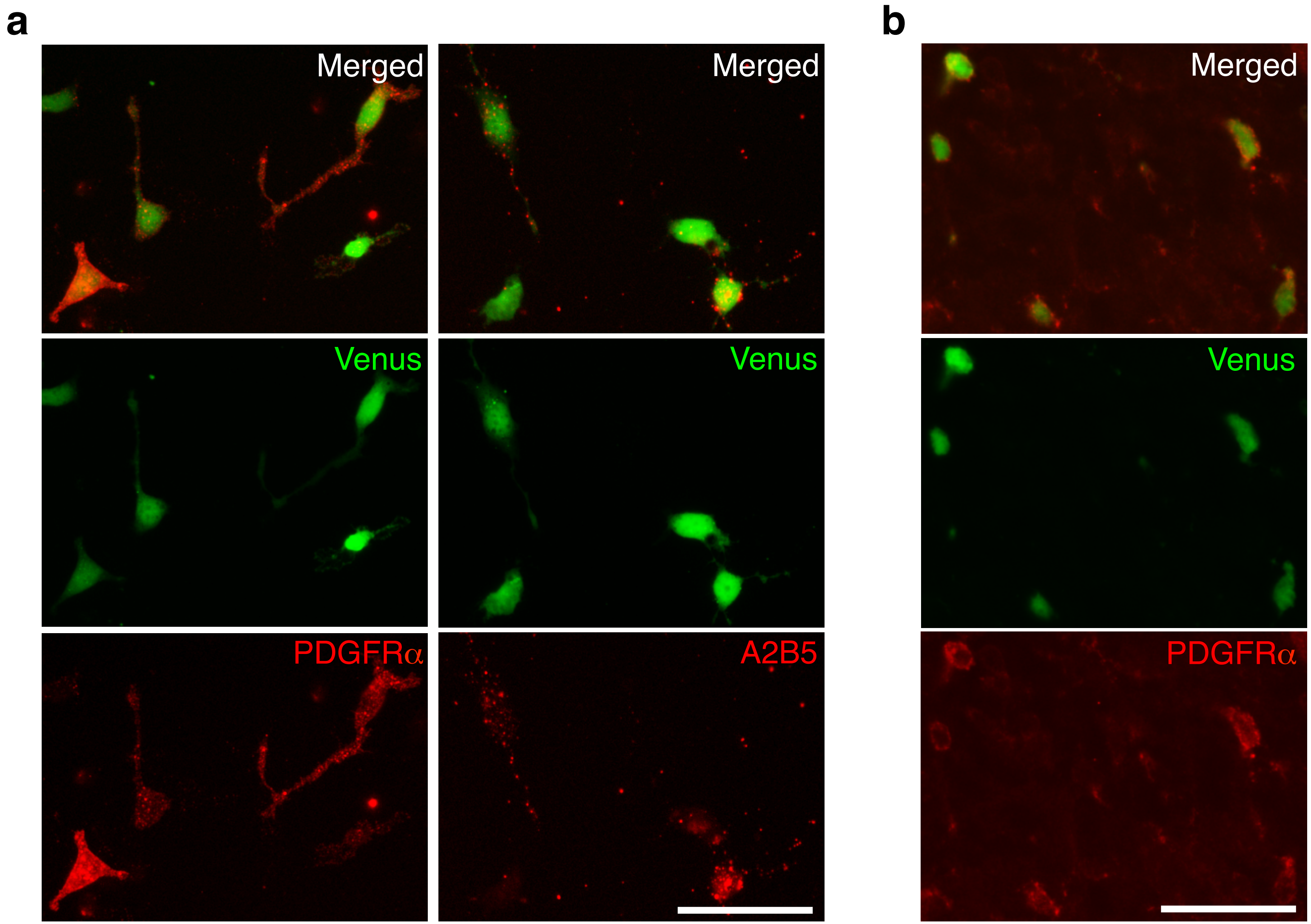

Supplementary Figure S2

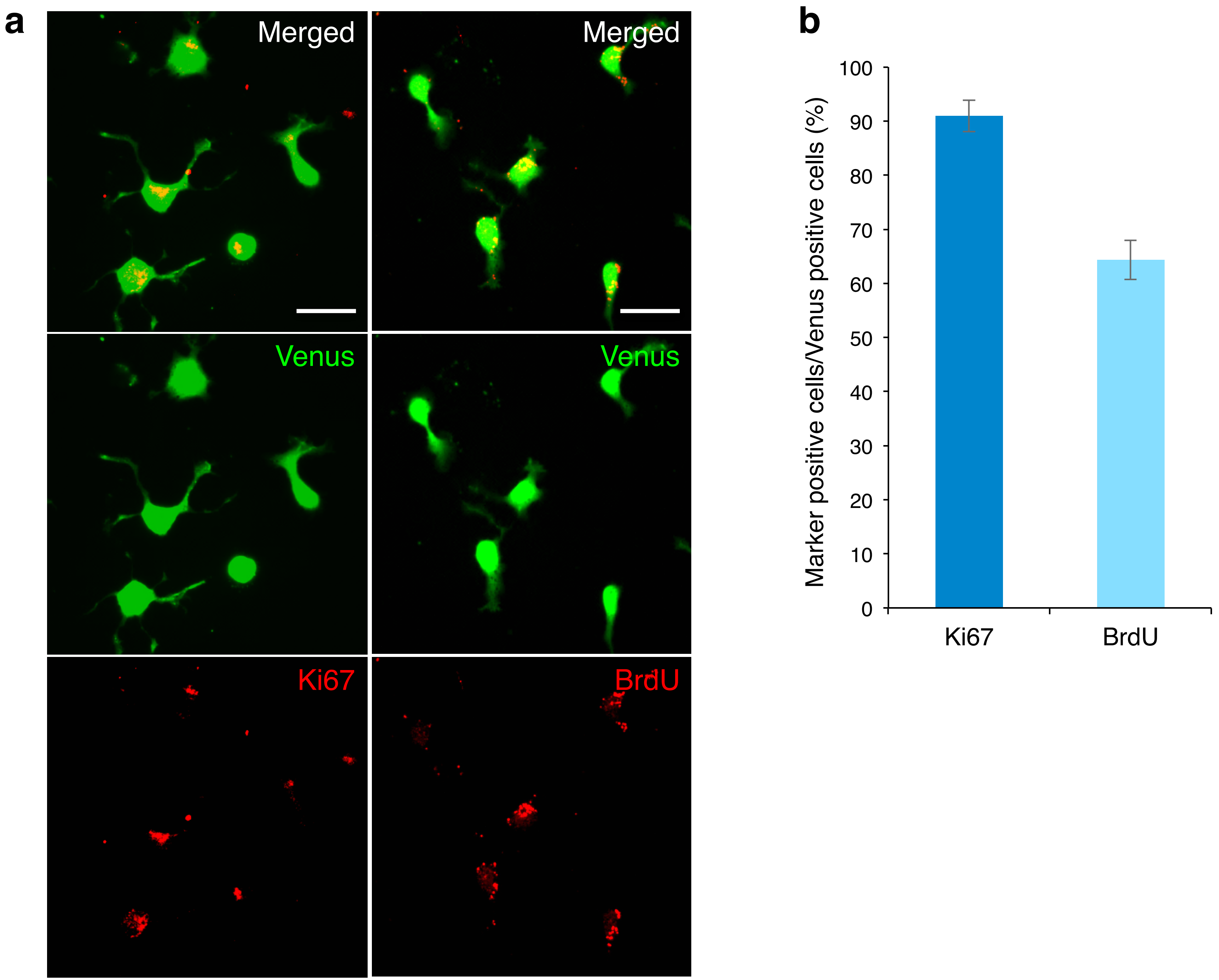

Supplementary Figure S3

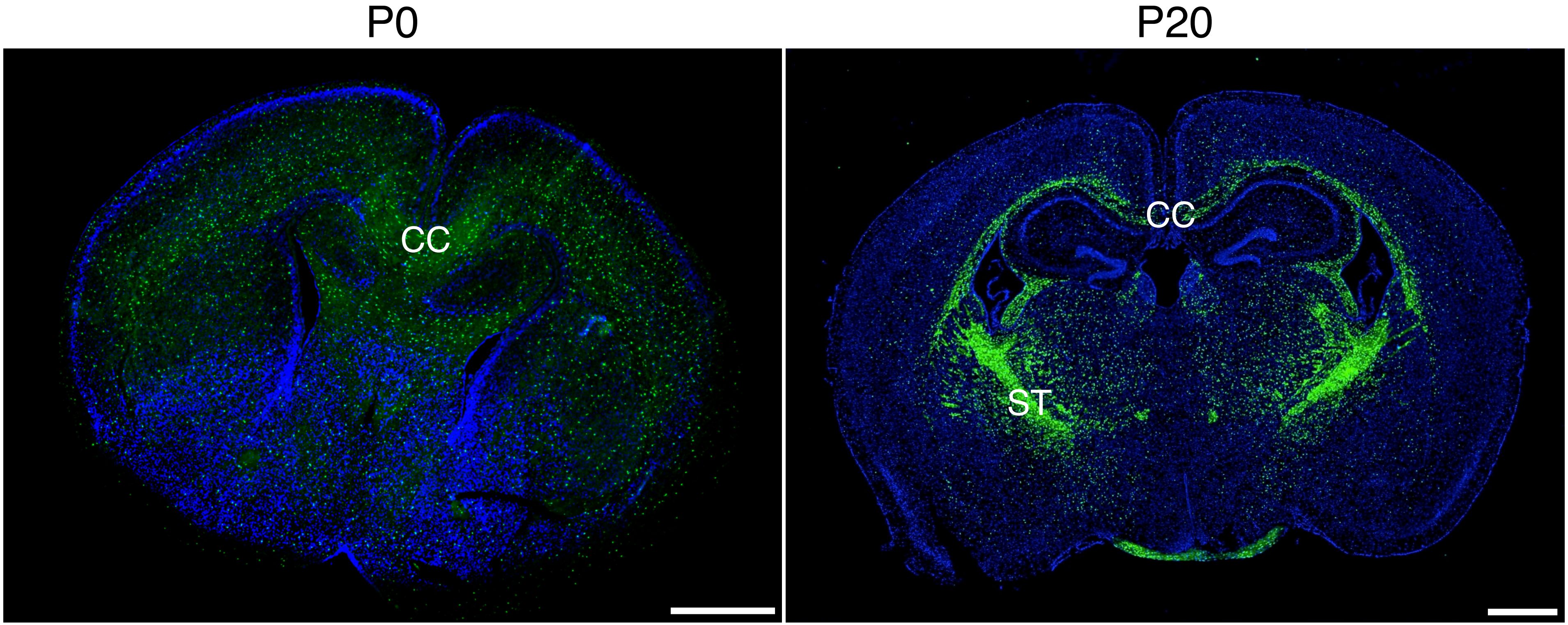

Supplement: Supplementary file 3 — Supplementary Figures [file 41598_2017_14207_MOESM3_ESM.pdf]
